# Prevention at work needed to curb the worldwide strong increase in knee replacement surgery for working-age osteoarthritis patients

**DOI:** 10.5271/sjweh.3915

**Published:** 2020-09-01

**Authors:** P. Paul F.M Kuijer, Alex Burdorf

**Affiliations:** Netherlands Center for Occupational Diseases, Department of Public and Occupational Health, Amsterdam Public Health Research Institute, Amsterdam Movement Sciences, Amsterdam UMC, University of Amsterdam, Amsterdam, The Netherlands. [e-mail: p.p.kuijer@amsterdamumc.nl]; Department of Public Health, Erasmus Medical Center, Rotterdam, The Netherlands [Email: a.burdorf@erasmusmc.nl]

In the upcoming decades, hospitals and clinics around the world face a steep rise in demand from patients seeking knee replacement surgery. An absolute increase in knee replacement surgery of 297% – to 57 893 procedures – is forecasted in The Netherlands between 2005 and 2030 (1). The situation is similar in many countries: Sweden, 163% to 21 700 (2013–2030) (2); Italy, 45% to about 100 000 (2017–2050) (3), the UK, 916% to about 1.2 million (2015–2035) (4); Australia, 276% to 65 569 (2013–2030) (5); and the USA, 673% to 3.48 million (2005–2030) (6). No projections are available for Asia, however, similar growth percentages have already been seen in Japan of 373% (2007–2014) (7) and Korea (407%, 2001–2010) (8). Knee replacement surgery or arthroplasty is the final treatment option for patients suffering from knee osteoarthritis (OA). These increasing numbers are alarming, not only due to the extreme high demands on healthcare provision and budgets, but also for the unforeseen impact on work participation. There is a lack of awareness how work might play a significant role in reducing the steep rise in replacement surgery for knee OA across the world.

**Unforeseen impact on work**

The largest increase in primary surgery demands is not among the classic knee arthroplasty population of patients aged 70 years and older, but among patients of working age (1–4, 9). For instance, Germany – one of the leading countries in the prevalence of knee arthroplasty – foresees the highest increase in patients aged 50– 65 years until 2050 (10), and in a similar study using the same database even among patients aged 40–49 years until 2040 (11). In several countries, the current proportion of knee arthroplasty patients under 65 years is already substantial at 30–40%. It is expected in 2030 that the USA will be the first country where the majority of these patients will be younger than 65 years (6), followed by the UK in 2035 (4).

This increase in surgery and shift towards younger age groups can largely be explained by good clinical and cost-effective long-term outcomes, no clear threshold for surgery, and the rising number of younger and more demanding knee OA patients (9). Originally, knee arthroplasty was mainly aimed at reducing pain, improving knee function and thereby enhancing the performance of activities of daily life in the patient population aged 70 years and older. The current success was totally unforeseen by LG Shiers, the founding father of knee arthroplasty (12). In 1954, he concluded: “… few surgeons will ever see 50 patients requiring arthroplasty, let alone operate on them, even in five years”. Nowadays, specialized orthopedic surgeons perform this procedure several times per day. Despite the good clinical outcomes, return to work is not that favorable after surgery. About two of every ten working-age patients are dissatisfied with their work ability due to their knee arthroplasty (13). The majority of workers return to their original or other work only after six months (13,14), and about three of every ten patients do not return to their original or other work after a year (13,15). Especially performance of knee-demanding activities like kneeling, crouching and clambering does not improve after surgery (16).

The rapid adaptation of knee arthroplasty as preferred clinical practice for working-age knee OA patients has profound consequences for functioning at work and labor force participation. These consequences receive little-to-no attention in current clinical guidelines and yet are increasingly important outcomes for patients of working age (17, 18). Therefore, occupational health professionals around the world face the urgent challenge to develop evidence of how to improve a patient’s ability for sustained employability – as an essential value in life – after surgery (19). Promising initiatives have recently been described although not validated for work participation yet (20–23). More importantly, occupational health professionals should play a primary role in reducing the steep increase in replacement surgery by mitigating the risk of knee OA.

**Work to reduce knee osteoarthritis and hospitalization**

Until now, little-to-no attention has been given to work as a promising point of engagement for the prevention of knee OA and subsequent hospitalization. A nationwide representative prospective Finnish study showed that high body mass index, prior knee injury, and an intermediate-to-high cumulative physical workload accounted for 70% of hospitalizations due to knee OA (23). Bearing these three modifiable risk factors in mind, preventive strategies at work seem warranted to reduce both the incidence of knee OA and, of course, the associated hospitalization. Primary prevention at work might mitigate all three risk factors. Without question, losing excessive body weight is key (23). Proper & Van Oostrom (24) concluded in their review on health promotion intervention, that strong evidence exists for favorable effects of weight reduction, especially for interventions targeting diet and/or physical activity. Regarding knee injury and cumulative physical workload, reducing the time kneeling and squatting at work appears highly promising to tackle both (25–27).

**Figure:**
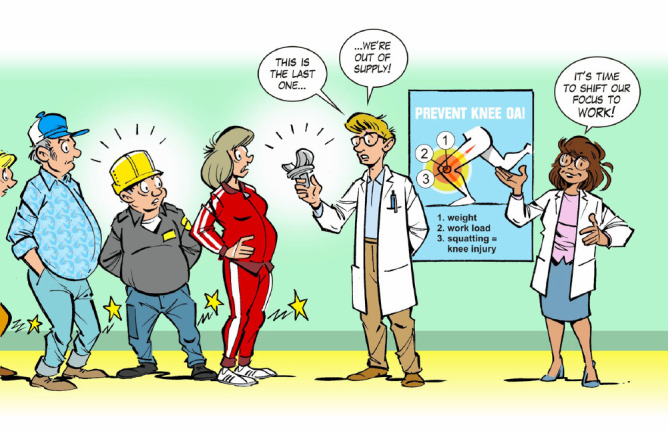
Working age knee osteoarthritis patients are especially at risk for knee replacement surgery despite the added value of work for prevention.

The upcoming EU-OSHA campaign 2020–2022 ‘Healthy Workplaces Lighten the Load’ starting October 2020 is an excellent opportunity to focus on this specific occupational disease. Intensive interventions tailored to the specific target group are promising to reduce the risk of knee OA (28-30). This approach is in line with the general risk assessment strategies to safeguard workers’ health and safety and, therefore, Labor Inspectorates can effectively enforce compliance with the occupational safety and health regulations (31). Moreover, the proposed combined interventions on lifestyle and workplace adaptations are also in line with the stepped-care strategy for secondary prevention of knee OA (32) and of course tertiary prevention by bringing the rising hospital admission curve to its knees and thereby contributing to better health and prolonged working lives of workers at risk of “running out of cartilage”.
